# PROFILES OF TWO COHORTS OF NEAR-CENTENARIANS AND CENTENARIANS: FINDINGS FROM HONG KONG CENTENARIAN STUDY 1 AND 2

**DOI:** 10.1093/geroni/igad104.1339

**Published:** 2023-12-21

**Authors:** Bobo Hi-po Lau

**Affiliations:** Hong Kong Shue Yan University, Hong Kong, Hong Kong

## Abstract

Hong Kong launched its first centenarian study in 2011 when there were just over 3,600 centenarians, and subsequently, the second phase in 2021/22 when the population of centenarians tripled. Both studies investigated the multidimensional well-being of local older adults of Chinese ethnicity aged 95 or above (near centenarians and centenarians, NCCs). A comparison of the two cohorts of community dwelling NCCs who lived with their family (2011: N=77; 2021/22: N=151) indicates a substantial drop in sensory functions (2011 vs 2021/22: intact vision: 94.8% vs 40.4%; intact hearing: 93.5% vs 53.6%), social activities (weekly or more: 48.1% vs 40.4%), outdoor activities (weekly or more: 75.3% vs 45.3%), and independence in activities of daily living (ADL) (no. of dependent task: 0.79 vs 2.79). Although the prevalence of comorbidities was comparable (Charlson age-adjusted comorbidity index: Mean = 2.7 vs 3.5), the frailty and pre-frailty was considerably more common in the 2021/22 cohort (55.3%; 39.0%) than the 2011 cohort (50.6%; 23.4%). We also found disparate predictors for health evaluations. Among the 2011 cohort, being male and having better psychological well-being were related to more favorable assessments of health compared to their peers. Among the 2021/22 cohort, favorable peer-compared health assessments were related to fewer ADL dependent tasks, better psychological well-being, higher age and being female. Despite the impact by COVID on the 2021/22 cohort, our findings alert policy makers and practitioners worldwide to the growing number of community-dwelling adults of advanced age with considerable personal care and health needs.

